# Supplementary material for: Structural basis of antiphage immunity generated by a prokaryotic Argonaute-associated SPARSA system
Source: Nat Commun. 2024 Jan 11;15:450. doi: 10.1038/s41467-023-44660-7 (PMC10781750; doi:10.1038/s41467-023-44660-7)
Supplement: Supplementary file 2 — Supplementary Information [file 41467_2023_44660_MOESM2_ESM.docx]

**Structural basis of antiphage immunity generated by a prokaryotic Argonaute-associated SPARSA system**

**
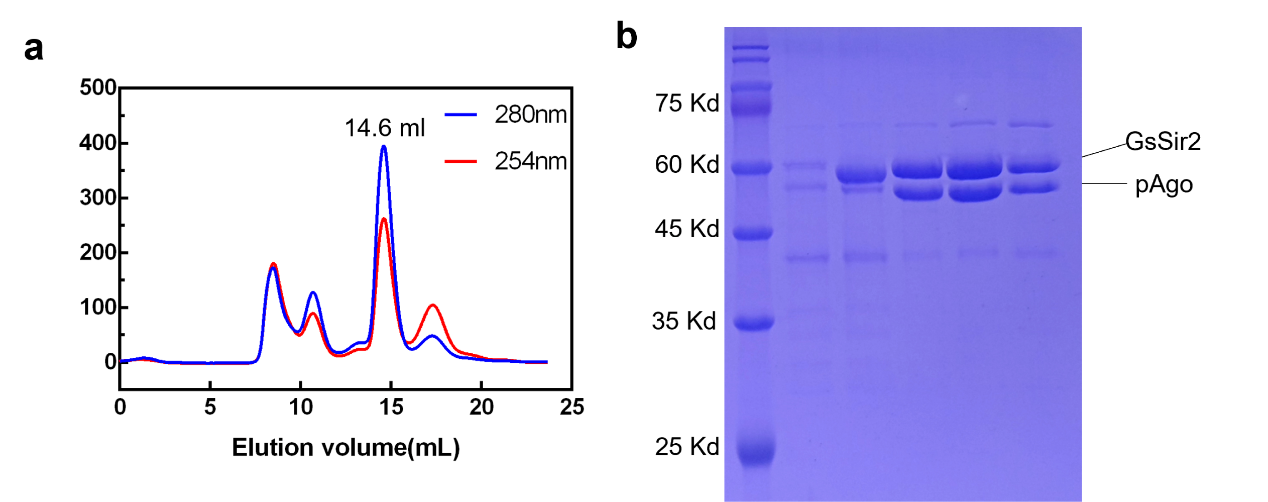
**

**Supplementary Fig. 1. Purification of the** **SPARSA heterodimeric complex.** (a), Representative size exclusion chromatography (SEC) of SPARSA, where the peak containing the target complex is illustrated by a black arrow. A volume of 14.6 mL of GsSir2-pAgo was eluted, and SDS-PAGE was performed to analyze its purity (a).

**
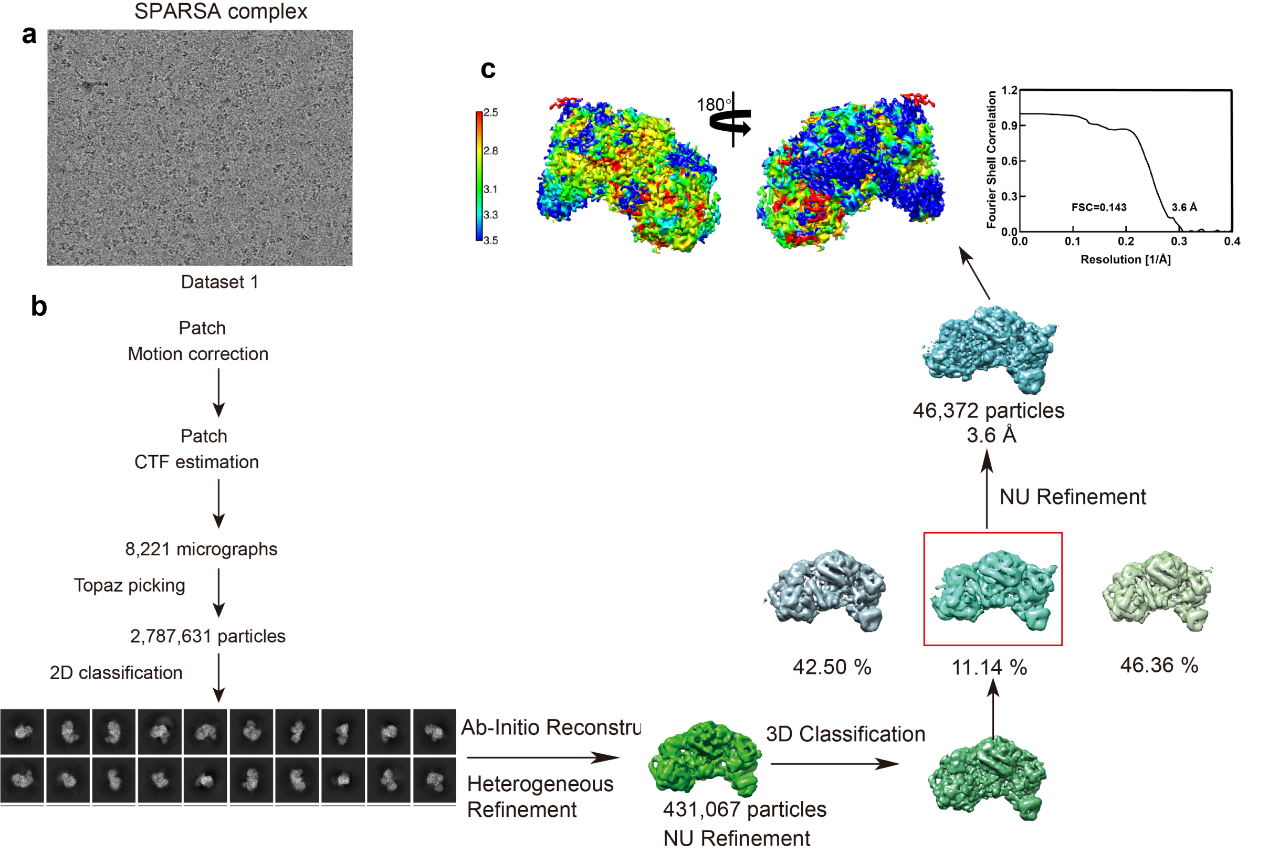
**

**Supplementary Fig. 2. Cryo-EM single particle analysis of the SPARSA binary complex**. (a) The representative cryo-EM micrograph images of the GsSir2-pAgo heterodimeric complex. (b) A flowchart of cryo-EM data processing. (c) The FSC curves between the cryo-EM density maps and the atomic modes of the SPARSA complex.

**
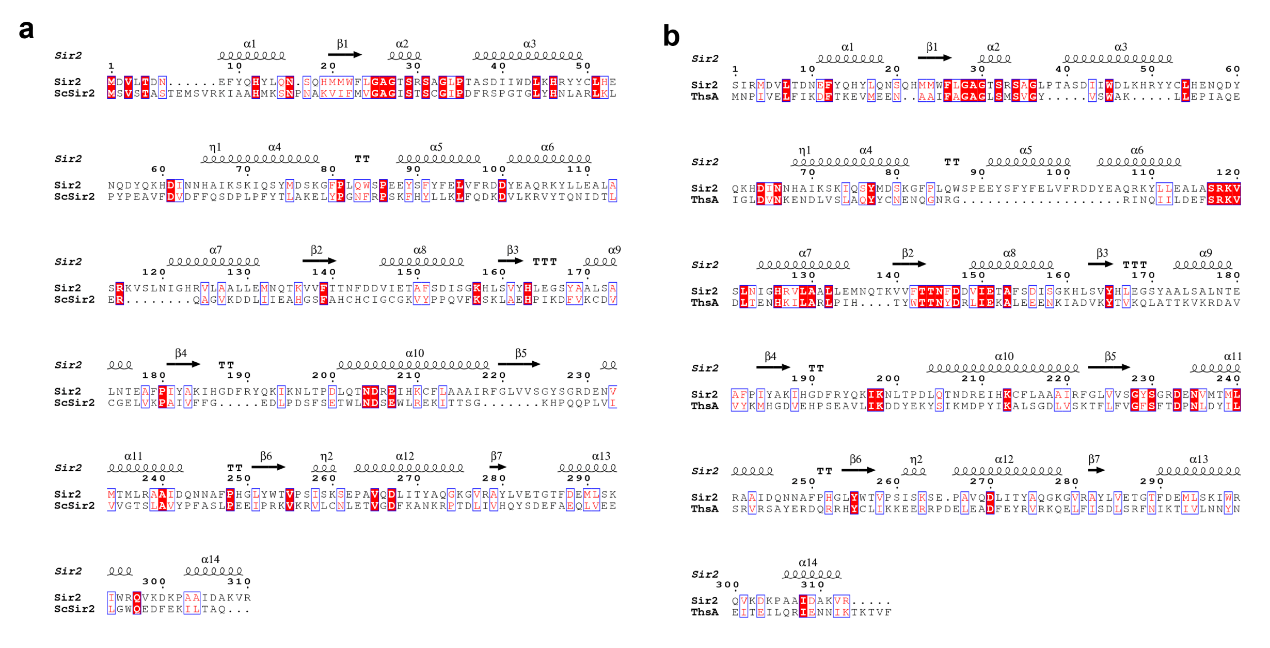
**

**Supplementary Fig. 3. Sequence alignment of the N-terminal Sir2 and the typical Sir2 proteins in prokaryotes** (a) and eukaryotes (b). The secondary structural elements of the Sir2 domain observed in the solved structure are shown at the top of the figure. The highly conserved residues are shown in red. The results showed a low sequence identity with ThsA and human Sir2 (SIRT2).

**
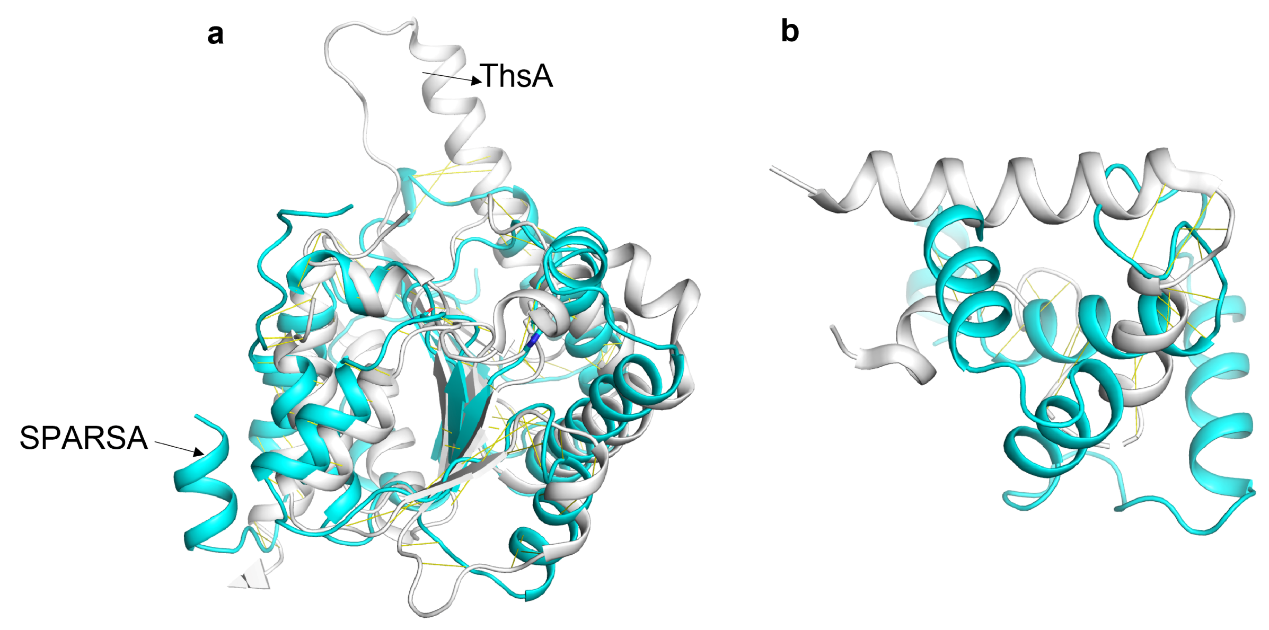
**

**Supplementary Fig. 4. Structural comparison of the Sir2 domain of SPARSA and the ThsA (PDB 6LHX).** (a) Superimposition of the Rossmann-like domain of Sir2 in SPARSA and ThsA that most of the core of the Rossmann domain can be aligned well. (b). There are large deviations in the small domain of the Sir2 in SPARSA and ThsA.

**
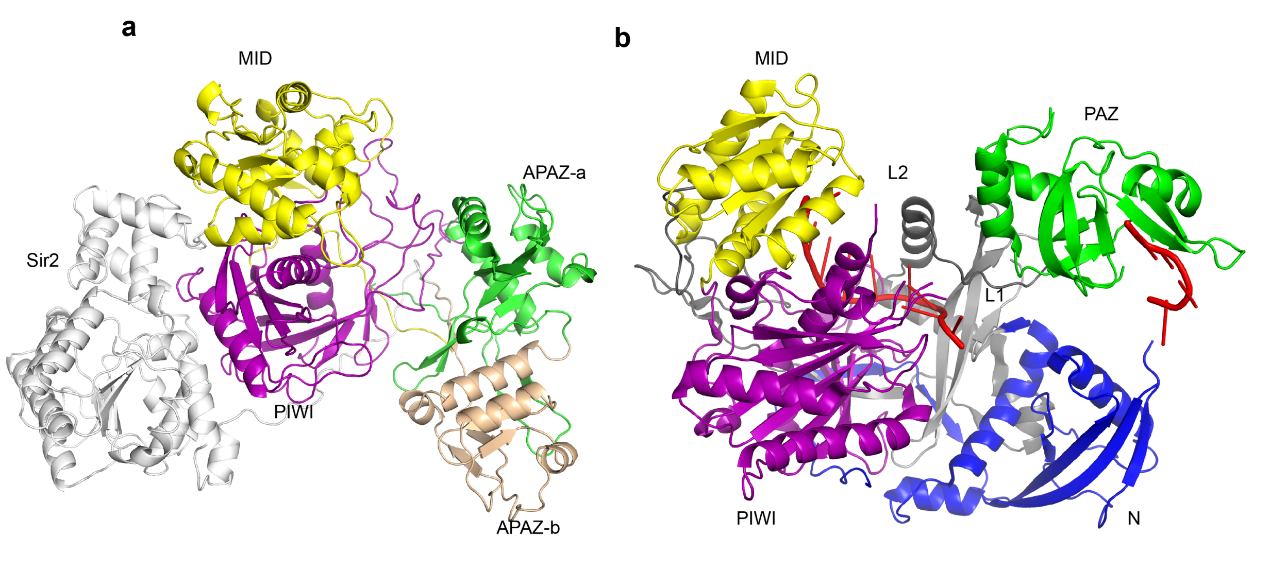
**

**Supplementary Fig. 5. The C-terminal two APAZ subdomains of GsSir2 correspond to the N and PAZ domains of the Ago-clade proteins.** (a) Structural highlights t the C-terminal APAZ subdomains of GsSir2, the two subdomains are colored in green and wheat (b) Structure of hAgos, the N and PAZ domains are colored in green and blue, and the gRNA is colored in red (PDB 4F3T).

**
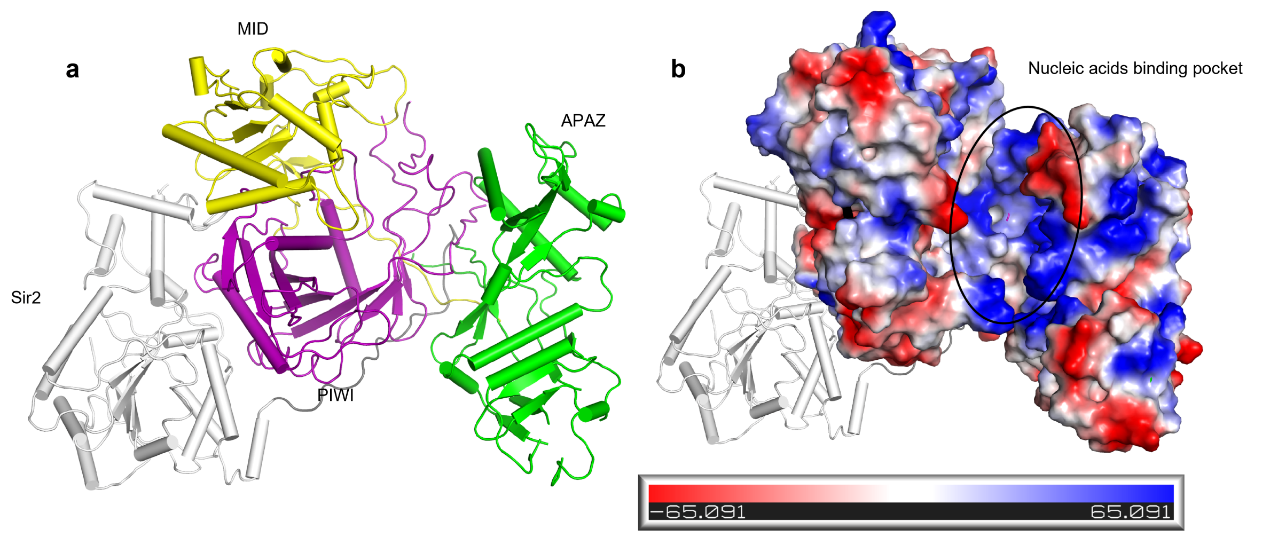
**

**Supplementary Fig. 6. The structure of pAgo and the APAZ domain was similar to the classical Ago-clade proteins**. (a) The structure of SPARSA, the N-terminal Sir2, is shown in white, the APAZ is shown in green, and the MID and PIWI domains of pAgo are shown in yellow and purple, respectively. (b) A positively charged tunnel is formed by the APAZ domain and pAgo.

**
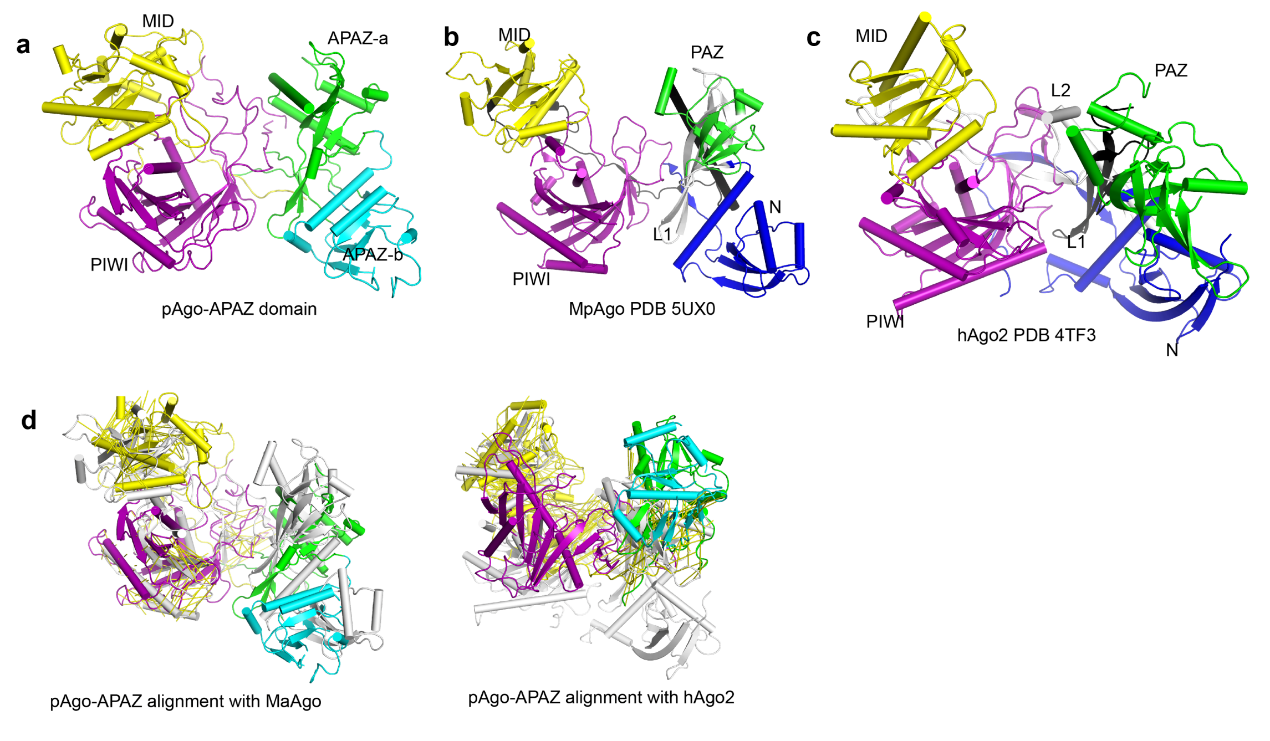
**

**Supplementary Fig. 7. The pAgo and the APAZ domain formed an architecture similar to the typical long Ago**. (a) The overall structure of pAgo-APAZ of *Geobacter sulfurreducens*. (b-c) The representative structures of pAgo (b) and eAgo (c). (d) Structural alignment of pAgo-APAZ and MaAgo (left panel) and hAgo2 (right panel).

**
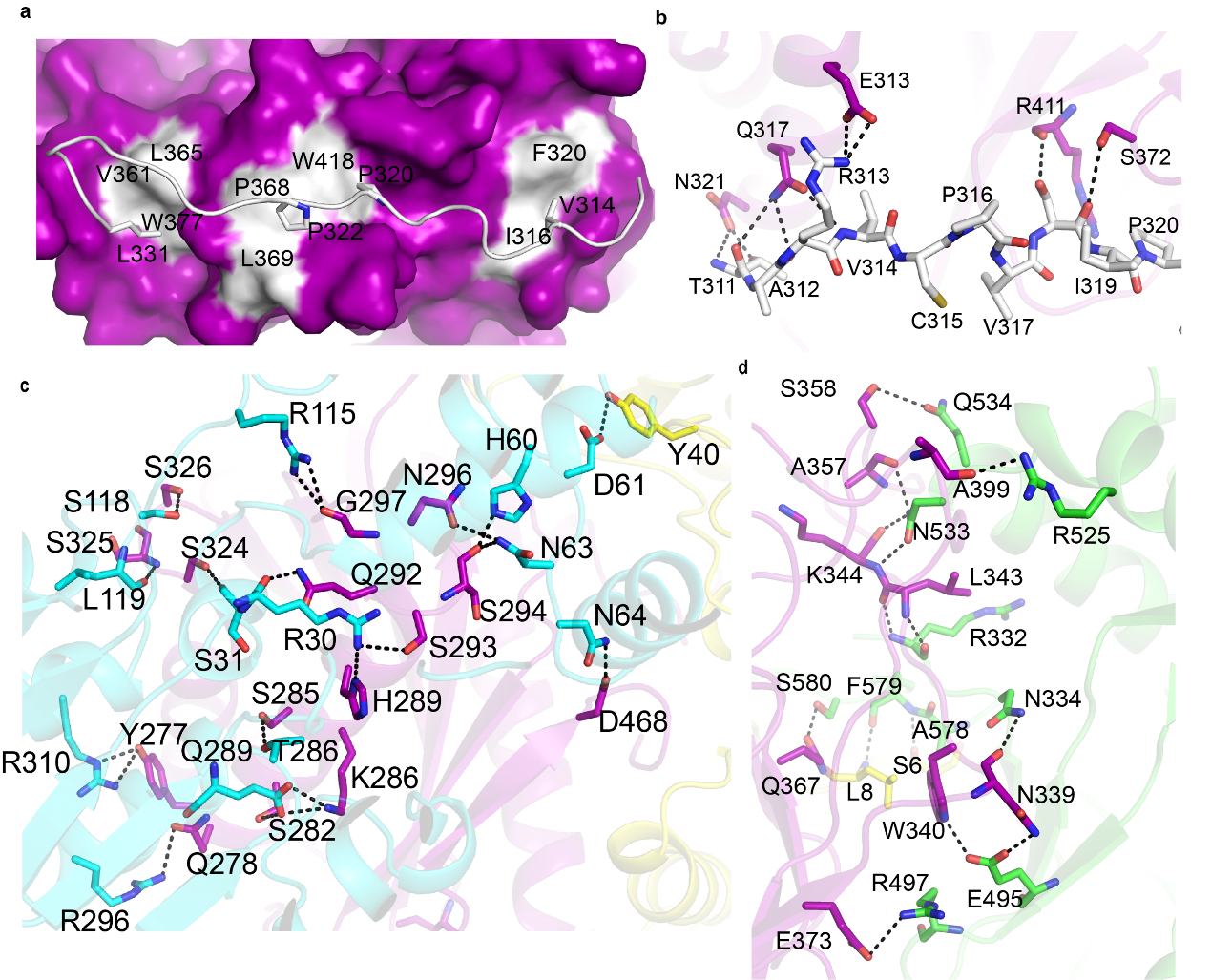
**

**Supplementary Fig. 8. The interaction between Sir2**-APAZ **and** p**Ago.** (a) Combined surface representation and a ribbon-stick model showing the molecular interface of the hydrophobic interactions between the loop and pAgo. The hydrophobic amino acid residues in the loop are shown as sticks, and the hydrophobic patch in the PIWI domain of pAgo is colored white. **(b)** The hydrogen-bond interactions between the loop and pAgo, the residues that are involved in the binding, are shown as sticks. **(c-d)** The molecular interaction between pAgo and the N-terminal Sir2 **(c)** domain and C-terminal APAZ domain **(d)** and the interactions involved are shown as sticks.


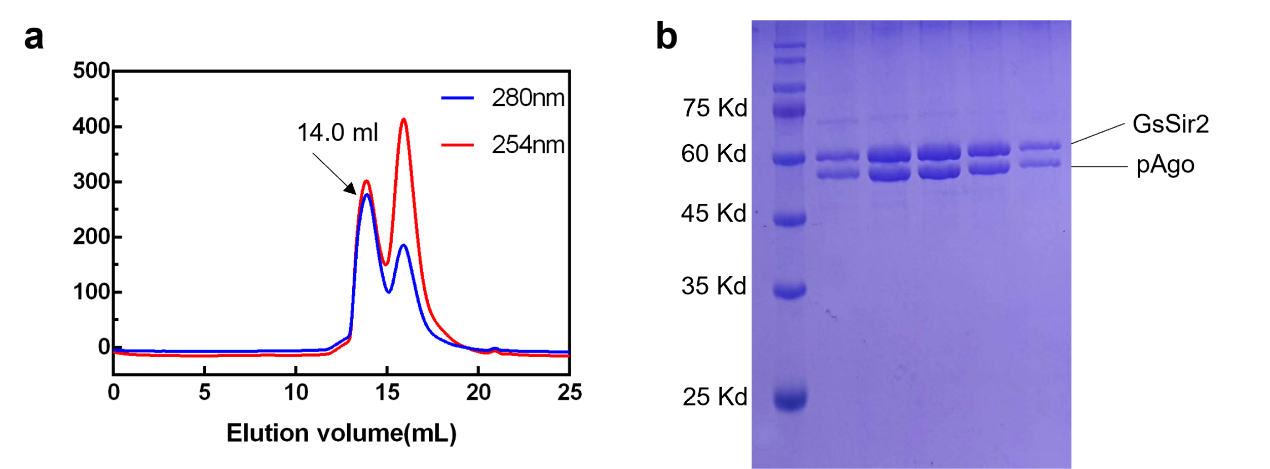


**Supplementary Fig. 9. The assembly of the SPARSA-gRNA-tDNA ternary complex**. The SEC elution profile of SPARSA-gRNA-tDNA (upper panel) and its SDS-PAGE analysis (bottom panel). It was purified using a process similar to that described in Extended Data Fig. 1.

**
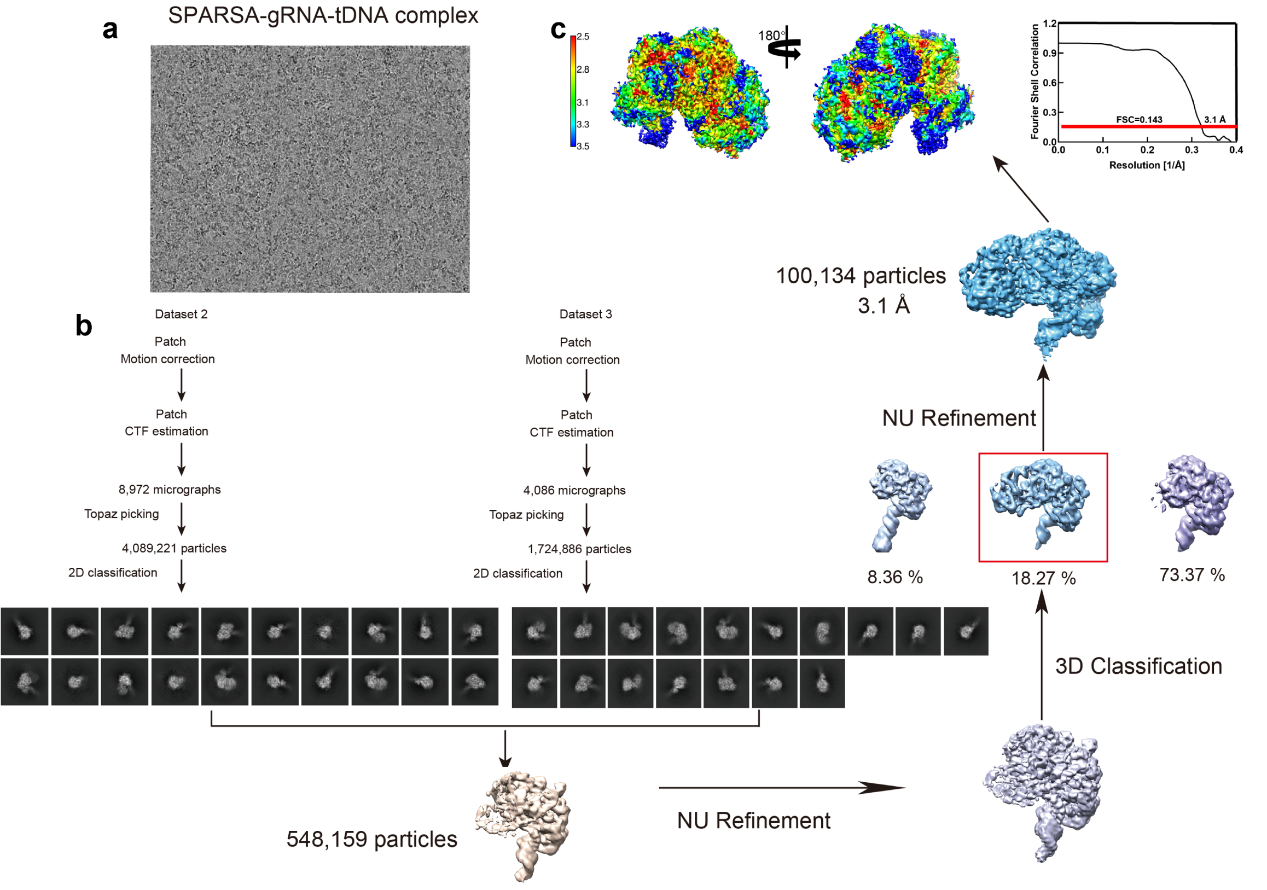
**

**Supplementary Fig. 10. Cryo-EM single-particle analysis of the GsSir2-sAgo-gRNA-tDNA complex**. (a) The representative cryo-EM micrograph images of the GsSir2-sAgo-gRNA-tDNA hetero-tetrameric complex. (b) A flowchart for cryo-EM data processing. (c) FSC curves between the cryo-EM density maps and the atomic modes of the GsSir2-sAgo-gRNA-tDNA complex.

**
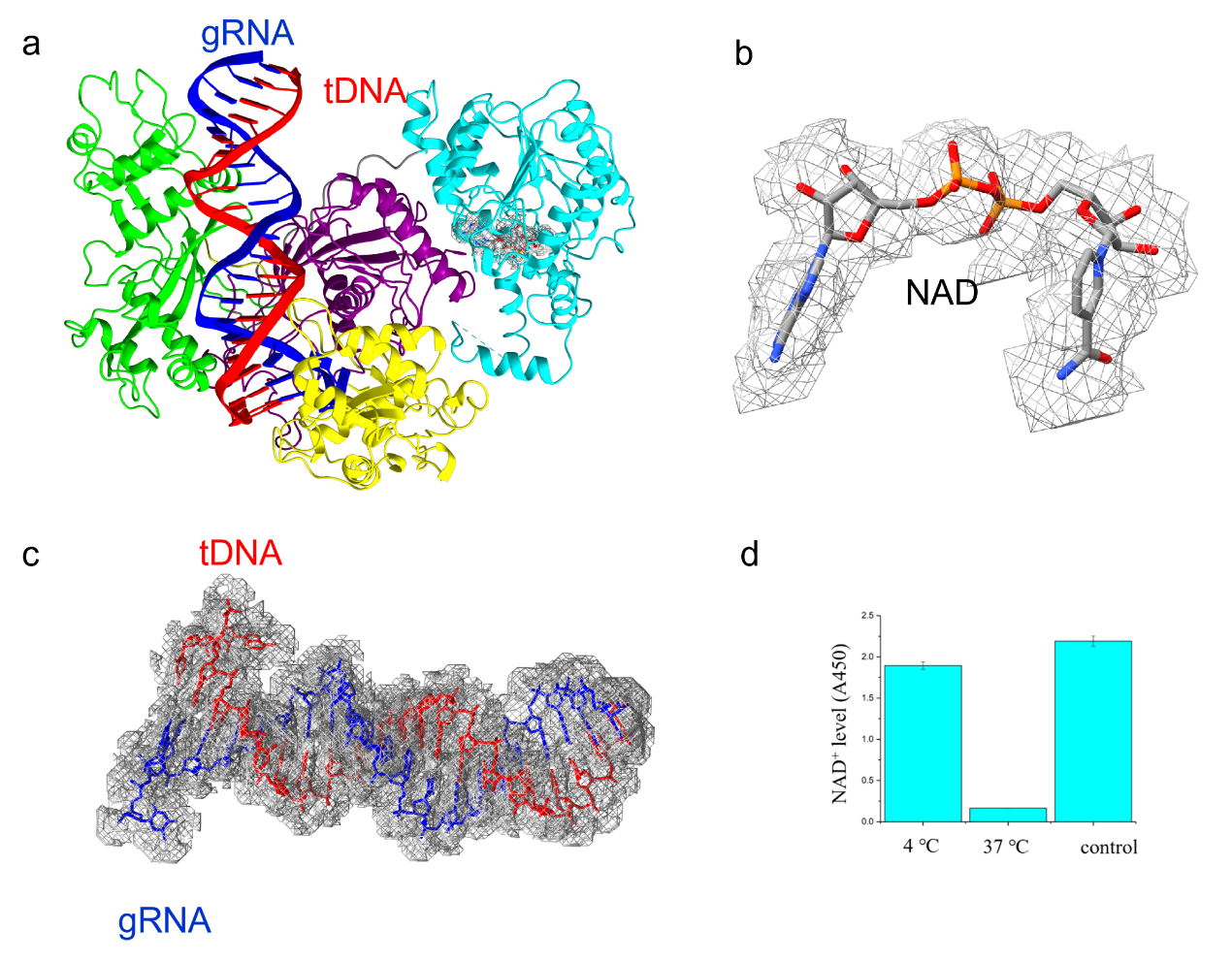
**

**Supplementary Fig. 11. Overall structure of SPARSA -gRNA-tDNA**. (a) The overall structure of SPARSA-gRNA-tDNA. (b) The density of SPARSA bound NAD**^+^**, the NAD**^+^** is shown in the sticks. (c) The cryo-EM density of NAD^+^. (d). The NADase activity of SPARSA-gRNA-tDNA is low at 4 °C during the process of assembly the SPARSA-gRNA-tDNA complex and cryo-EM data collection. The lanes represent the the results of control, 4 °C and 37 °C, respectively. Each value is the mean ± SD and from more than three replicates.

**
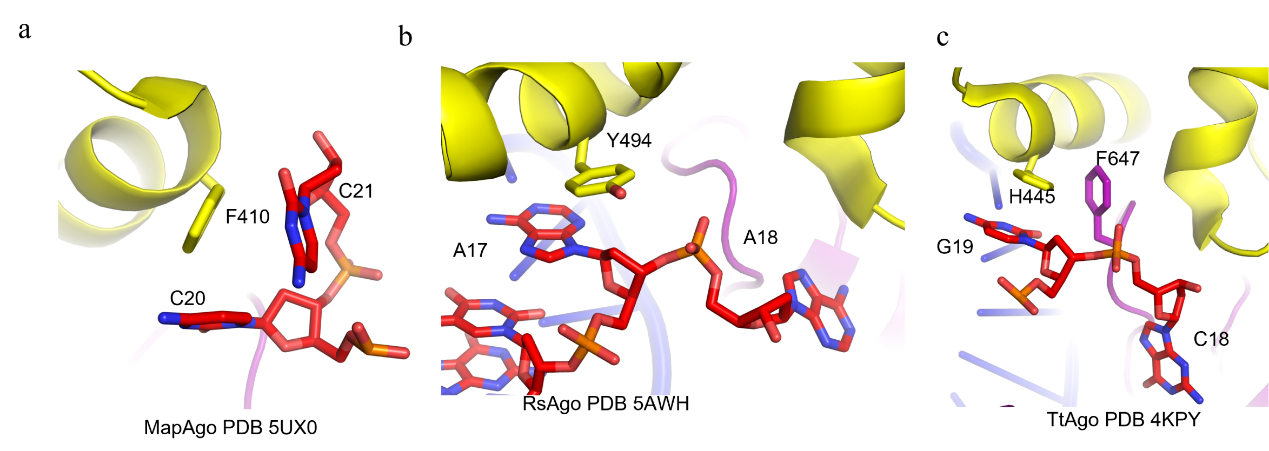
**

**Supplementary Fig. 12. The known target-bound Ago with the 3' terminal base of tDNA flipped away from gRNA**. (a) F410 of the MID domain inserted between C20 and C21 of the tDNA (MapAgo PDB 5UXO), (b) Y494 of the MID domain of RsAgo inserted between A17 and A18 of the tDNA (PDB 5AWH), (c) H445 and F467 of the MID domain inserted between C18 and G19 of the tDNA (TtAgo PDB 4CNB)

**
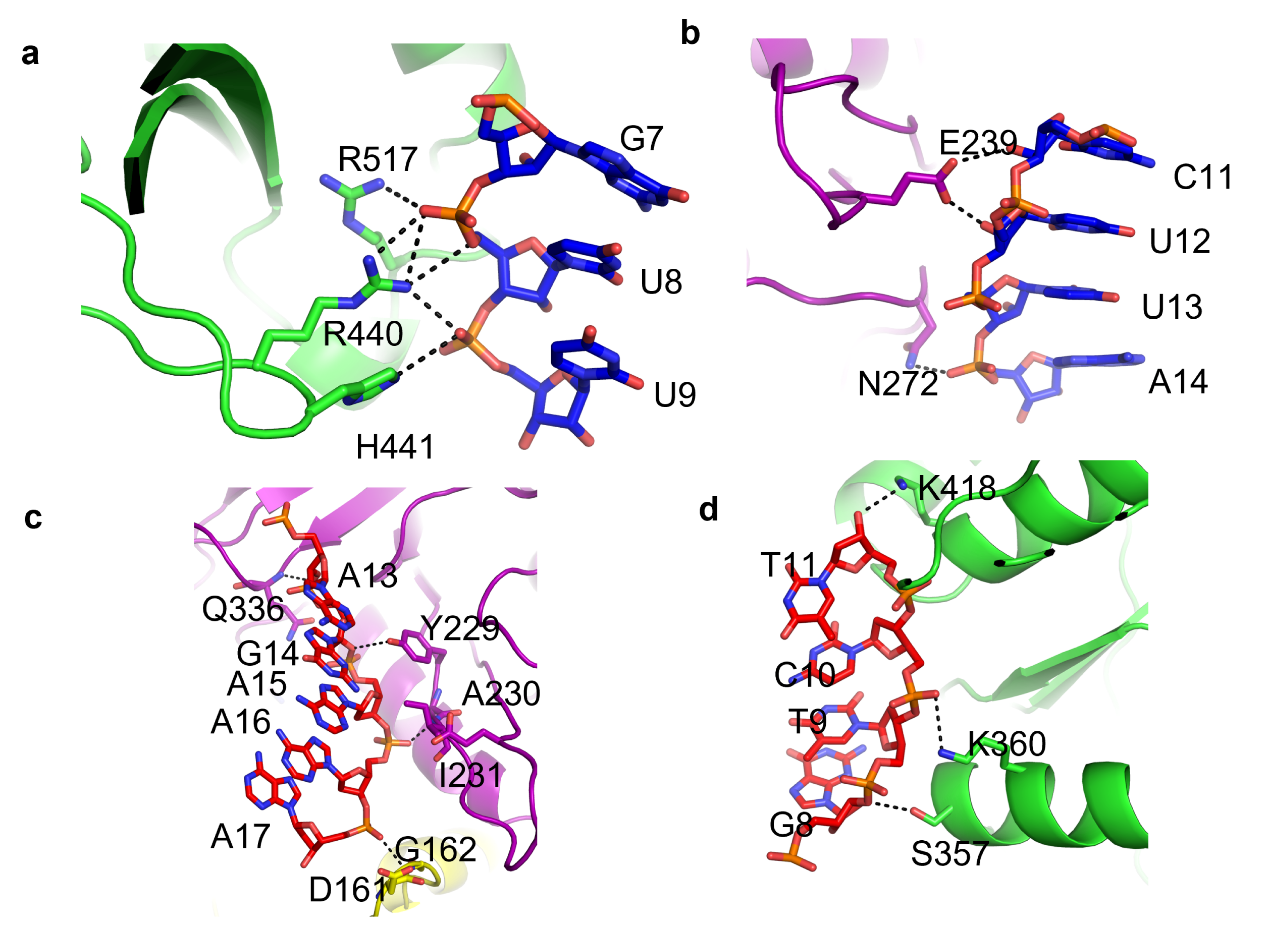
**

**Supplementary Fig. 13. The interactions between SPARSA and gRNA and tDNA**. The intermolecular contacts between the sugar-phosphate backbone of the gRNA (a-b) and tDNA (c-d), the gRNA and tDNA, are shown in the sticks and represented in blue and red, respectively; the residues that bind are shown in the sticks.

**
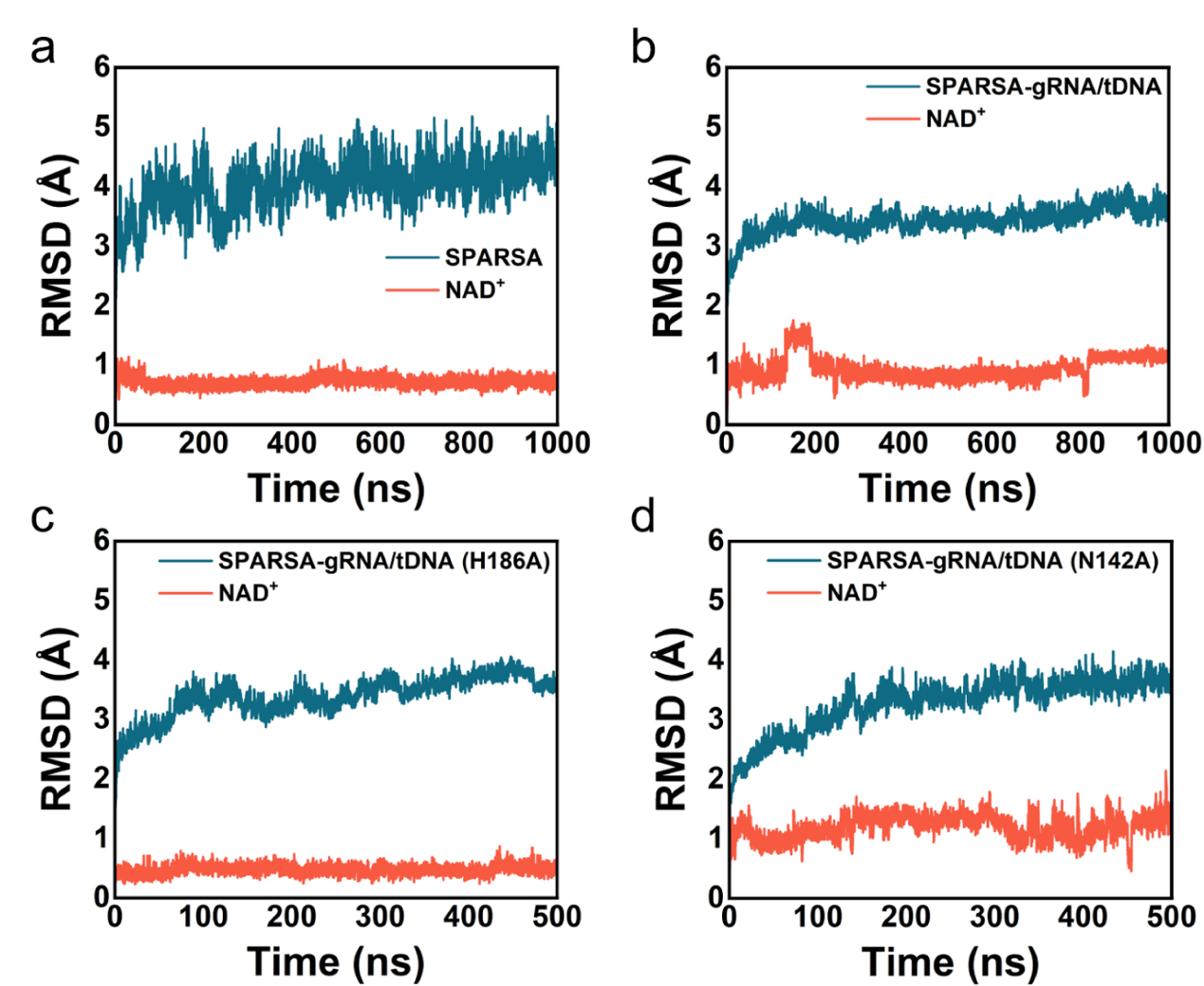
**

**Supplementary Fig. 14. Time evolution of the RMSD of** **SPARSA and SPARSA-gRNA/tRNA backbone atoms and NAD^+^ heavy atoms in the MD simulations of SPARSA /NAD^+^.** **(a)**, SPARSA-NAD^+^, **(b)** SPARSA-gRNA/tDNA/NAD^+^, **(c)** GsSir2/pAgo-gRNA/tDNA/NAD^+^ with single mutation of H186A, **(d)** SPARSA -gRNA/tDNA/NAD^+^ with single mutation of N142A .

**
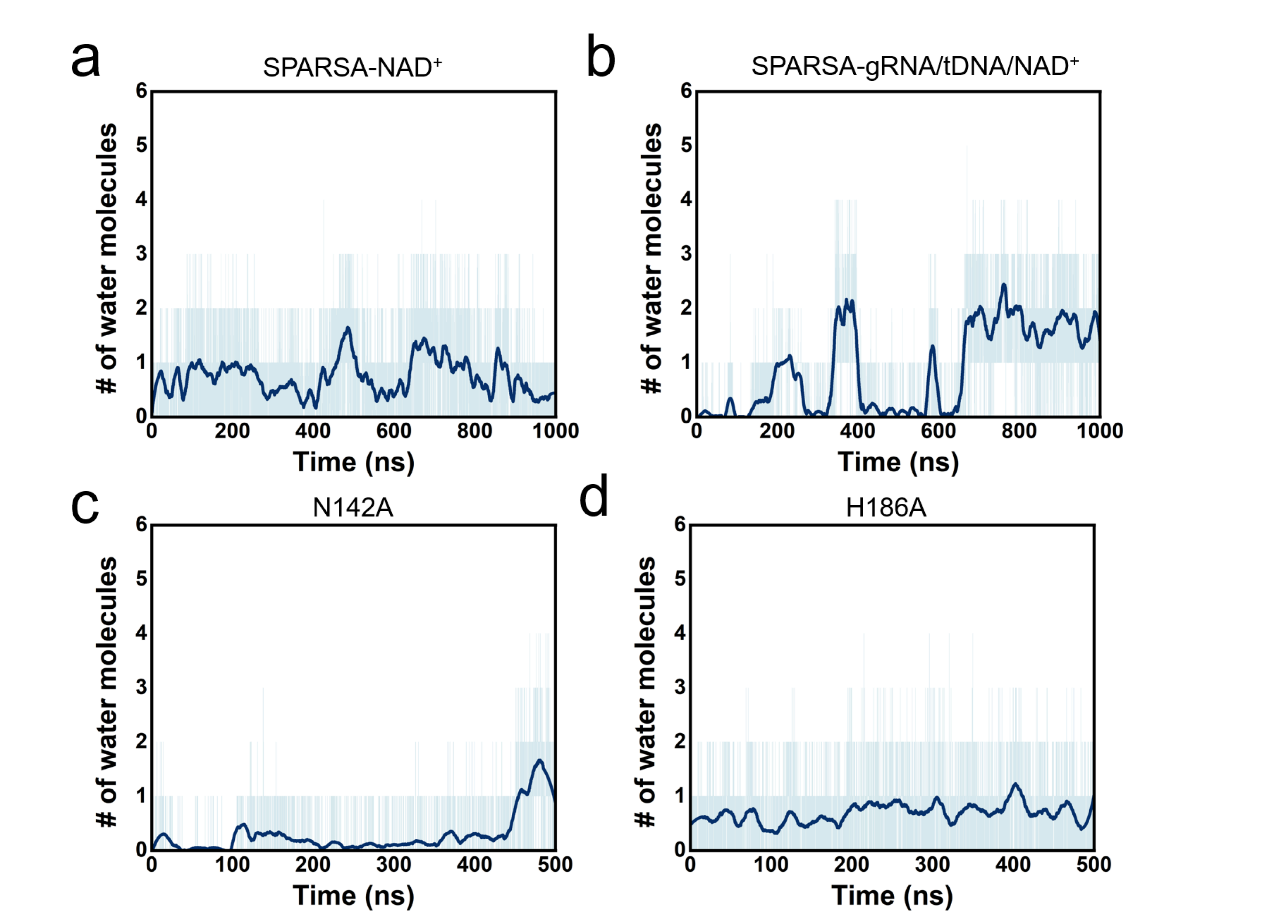
**

**Supplementary Fig.15. Time evolution of the number of water molecules around C1' atom of NAD^+^ within a cutoff of 3.5 Å during MD simulations. (a)** SPARSA/NAD^+^, **(b)** SPARSA-gRNA/tDNA/NAD^+^, **(c)** SPARSA-gRNA/tDNA/NAD^+^ with single mutation of N142A, **(d)** SPARSA -gRNA/tDNA/NAD^+^ with single mutation of H186A .

**
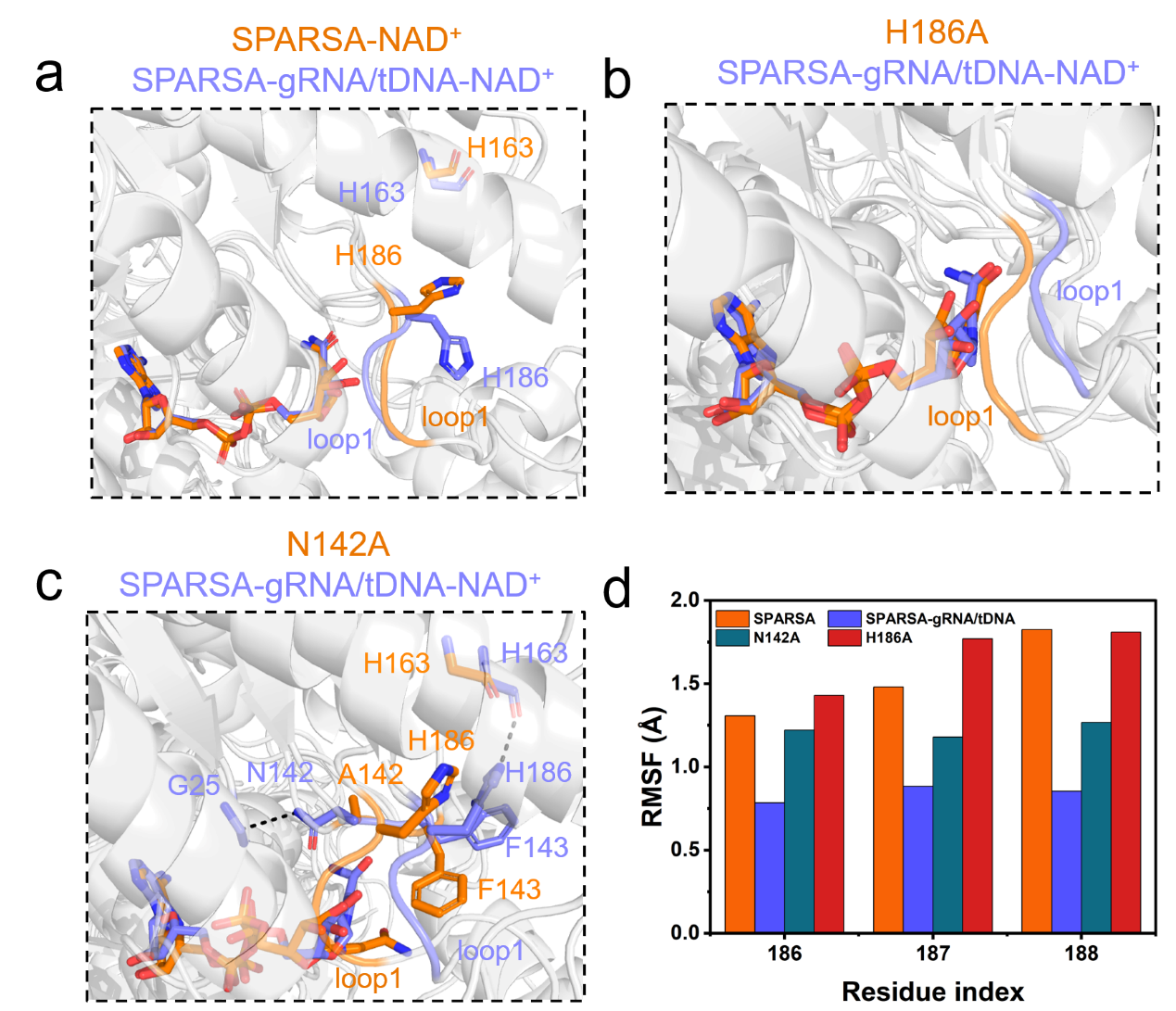
**

**Supplementary Fig.16. The remarkable conformational change of loop1 (^186^HGD^188^) mediated by the hydrogen bond interaction between H186 and H163. (a)** The position of the loop1 before MD simulations in SPARSA-NAD^+^ and SPARSA-gRNA/tDNA/NAD**^+^**, **(b)** and after MD simulations in SPARSA-gRNA/tDNA/NAD^+^ with single mutation of H186A, **(c)** and N142A . **(d)** The RMSF of the loop1 residues .

**
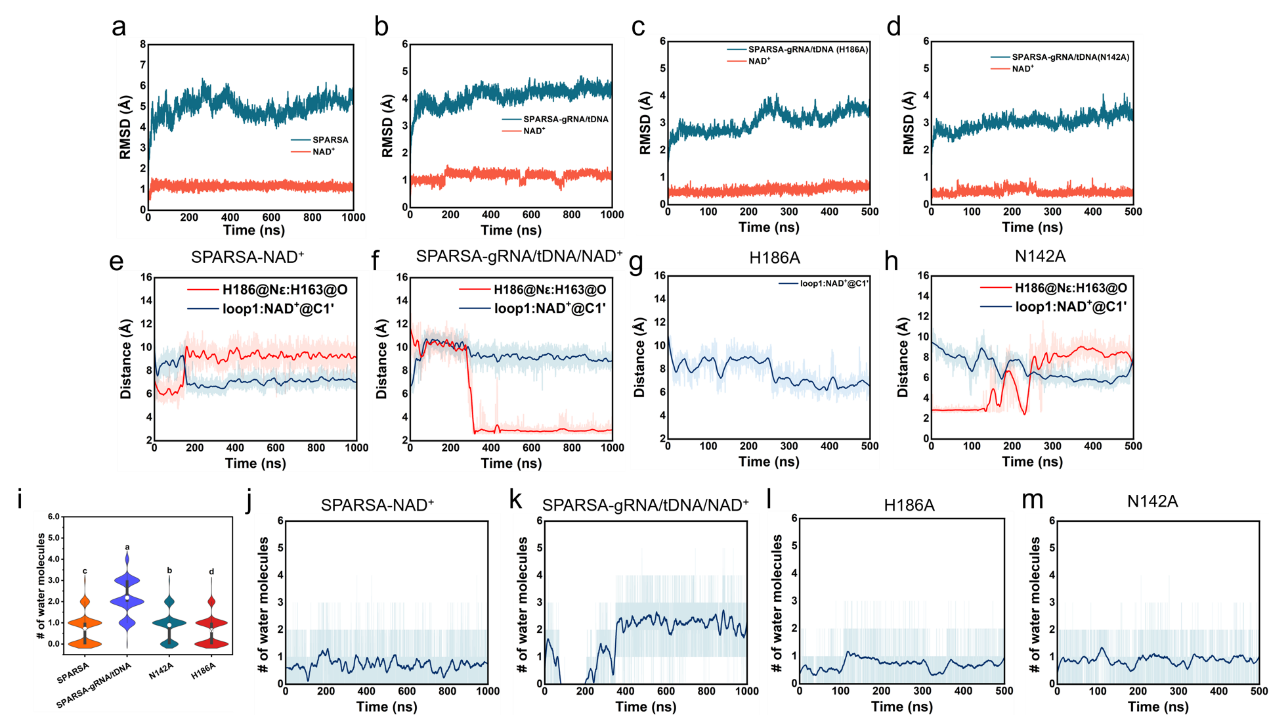
**

**Supplementary Fig.17. The replica MD simulations of SPARSA and SPARSA-gRNA/tRNA.** Time evolution of the RMSD of SPARSA and SPARSA-gRNA/tRNA backbone atoms and NAD^+^ heavy atoms in the MD simulations of SPARSA/NAD^+^ **(a-d)**. The distance between the Nε atom of H186 and the O atom of H163 and the distance between loop1 and C1' atom **(e-h)**. The number of water molecules around C1' atom of NAD^+^ within a cutoff of 3.5 Å **(i-m)**. The statistical significance was analyzed by using one-way ANOVA followed by Bartlett test. A *P* value of less than 0.05 was considered statistically significant.

**Supplementary Table 1**

Data collection and processing statistics of GsSir2/pAgo without and with gRNA/tDNA

|  |  |  |
| --- | --- | --- |
|  | SPARSA  PDB:8JKZ | SPARSA RNA/DNA  PDB:8JL0 |
| Data collection and processing |  |  |
| Magnification | 105,000 | 105,000 |
| Voltage(kV) | 300 | 300 |
| Electron exposure(e^-^/Å^2^) | 50 | 50 |
| Defocus range(μm) | -1 to -2 | -1 to -2 |
| Pixel size(Å) | 0.84 | 0.84 |
| Symmetry imposed | C1 | C1 |
| Initial particle images(no.) | 861,903 | 705,887 |
| Final particle image(no.) | 46,372 | 100,134 |
| Map resolution(Å) | 3.6 | 3.1 |
| FSC threshold | 0.143 | 0.143 |
| Refinement |  |  |
| Initial model used (PDB code) | -108 | -80 |
| Map sharpening *B* factor (Å^2^) |  |  |
| Model composition |  |  |
| Protein residues | 1053 | 1041 |
| Nucleic Acid | 0 | 22 |
| Ligand | 1 | 1 |
| Validation |  |  |
| Module Map scores |  |  |
| CC (correlation coefficients) | 0.84 | 0.85 |
| Average FSC (0/0.143/0.5) | 3.1/3.2/3.5 | 2.5/2.6/3.1 |
| R.m.s. deviations |  |  |
| Bond lengths(Å) | 0.003 | 0.004 |
| Bond angles(°) | 0.677 | 0.669 |
| MolProbity score | 2.07 | 1.97 |
| Clashscore | 9.82 | 8.63 |
| Rotamer outliers (%) | 1.00 | 0.79 |
|  |  |  |
| Ramachandran plot |  |  |
| Favored (%) | 89.90 | 91.31 |
| Allowed (%) | 10.01 | 8.30 |
| Disallowed (%) | 0.10 | 0.39 |

**Supplementary Table 2**

DALI search results against the PDB using the N-terminal Sir2 domain (The first five are listed)

| **No.** | **PDB ID-chain number** | **Z Score** | **RMSD** | **% Identity** | **Description** |
| --- | --- | --- | --- | --- | --- |
| **1** | 7UXT-A | 17 | 4.0 | 15 | ThsA in the Theoris |
| **2** | 5Y2F-A | 11.9 | 3.5 | 12 | NAD-dependent protein deacetylases Sir6 |
| **3** | 4IAO-B | 11.3 | 3.1 | 15 | NAD-dependent protein deacetylases Sir2 |
| **4** | 5A3A | 10.3 | 3.3 | 12 | Sir2 family proteins |
| **5** | 6LRG | 9.5 | 3.6 | 8 | Arginine-guanidine removing enzyme |

**Supplementary Table 3**

The primers used in this study.

| **Primers** | **Sequence** |
| --- | --- |
| Sir2F532A-F | AGTCGAAGACGTTATCGAGCCAATCAATGTTCATAT |
| Sir2F532A-R | CTGATATGAACATTGATTGGCTCGATAACGTCTTCG |
| pAgo-Y84A-F | AAGGAAGCGACGAACTATGCTGTGGAGTATGGAGGA |
| pAgo-Y84A-R | AAATCCTCCATACTCCACAGCATAGTTCGTCGCTTC |
| Sir2R30A-F | TTTAGGGGCAGGAACGTCTGCTTCTGCTGGTTT |
| Sir2R30A-R | AGTAGGCAAACCAGCAGAAGCAGACGTTCCTGC |
| Sir2S38A-F | GCTGGTTTGCCTACTGCTGCCGATATCATTTGG |
| Sir2S38A-R | AAGATCCCAAATGATATCGGCAGCAGTAGGCAA |
| Sir2F143A-F | GTTGTTTTCACAACCAACGCTGATGATGTCATA |
| Sir2F143a-R | GGTTTCTATGACATCATCAGCGTTGGTTGTGAA |
| Sir2R190a-F | CAAGATTCATGGCGACTTCGCTTATCAAAAAAT |
| Sir2R190a-r | ATTTTTTATTTTTTGATAAGCGAAGTCGCCATG |
| Sir2F189a-F | GCCAAGATTCATGGCGACGCCCGTTATCAAAAA |
| Sir2F189a-R | TTTTATTTTTTGATAACGGGCGTCGCCATGAAT |
| sir2-S227a-F | GTATCAGGCTATGCTGGTCGTGATGAAAACGTT |
| Sir2S227a-R | TTCATCACGACCAGCATAGCCTGATACAACAAG |
| Sir2Y226a- F | GGTCTTGTTGTATCAGGCGCTAGTGGTCGTGAT |
| Sir2Y226a-R | GTTTTCATCACGACCACTAGCGCCTGATACAAC |
| Sir2E262a-F | CCCTCTATTTCTAAGTCGGCGCCAGCCGTTCAG |
| Sir2E262a-R | CAAATCCTGAACGGCTGGCGCCCGACTTAGAAAT |
| Sir2F287a-F | CTGGTGGAAACCGGTACAGCTGATGAAATGCTC |
| Sir2F287a-R | CTTTGAGAGCATTTCATCAGCTGTACCGGTTTC |
| pAgoK156E-F | TTGCCTGCCAGCTGGAAGGAGTGTTTTGAGTATGAT |
| pAgoK156E-R | TCCATCATACTCAAAACACTCCTTCCAGCTGGCAGG |
| pAgoD164A-F | TTTGAGTATGATGGATTTGCCCTGCATGACCGA |
| pAgoD164A-R | CTTTATTCGGTCATGCAGGGCAAATCCATCATA |
| pAgoH166A-F | TATGATGGATTTGACCTGGCTGACCGAATAAAGGCG |
| pAgoH166A-R | TTTCGCCTTTATTCGGTCAGCCAGGTCAAATCCATC |
| pAgoK170E- F | GACCTGCATGACCGAATAGAGGCGAAAGTTGCTCCG |
| pAgoK170E-R | GAGCGGAGCAACTTTCGCCTCTATTCGGTCATGCAG |
| pAgoQ181A-F | CCGCTCAATTTGCCTATTGCAATCATCAACGACACT |
| pAgoQ181A-R | CGCAGTGTCGTTGATGATTGCAATAGGCAAATTGAG |
| pAgoN184A-R | TCTCGTTAGCGCAGTGTCGGCGATGATTTGAAT |
| pAgoN184A- F | TTGCCTATTCAAATCATCGCCGACACTGCGCTA |
| pAgoR190A- F | AACGACACTGCGCTAACGGCACAATGCCGCGCCAAT |
| pAgoR190A-R | GACATTGGCGCGGCATTGTGCCGTTAGCGCAGTGTC |
| pAgoN195A-R | GCTGACACCCCACATGACAGCGGCGCGGCATTGTCT |
| pAgoN195A-F | ACGAGACAATGCCGCGCCGCTGTCATGTGGGGTGTC |
| pAgoY229A-F | AGCTTATATCGGACTCAGTGCTGCCATCAAGAAAAA |
| pAgoY229A-R | AGCATTTTTCTTGATGGCAGCACTGAGTCCGATATA |
| pAgoE239A-F | AAAAATGCTGAAGGACAAGCATATACAACGTGCTGC |
| pAgoE239A-R | GCTGCAGCACGTTGTATATGCTTGTCCTTCAGCATT |
| pAgoN434A-F | CTGACGAAGGTTGACTGGGCCAATAATACGCTCTAC |
| pAgoN434A-R | TTTGTAGAGCGTATTATTGGCCCAGTCAACCTTCGT |
| pAgoN436A-F | AAGGTTGACTGGAACAATGCTACGCTCTACAAAAAG |
| pAgoN436A-R | TAACTTTTTGTAGAGCGTAGCATTGTTCCAGTCAAC |
| pAgoT437A-F | GTTGACTGGAACAATAATgCGCTCTACAAAAAGTTA |
| pAgoT437A-R | TGGTAACTTTTTGTAGAGCGCATTATTGTTCCAGTC |
| pAgoK440a- F | AACAATAATACGCTCTACGCAAAGTTACCAGTCACT |
| pAgoK440A-R | GAGAGTGACTGGTAACTTTGCGTAGAGCGTATTATT |
| pAgoK441A-F | AATAATACGCTCTACAAAGCGTTACCAGTCACTCTC |
| pAgoK441A-R | AACGAGAGTGACTGGTAACGCTTTGTAGAGCGTATT |
| Sir2R517A-F | ATAAAGCCATTGGATAGGGCAGAAGAGGCCACGGAT |
| Sir2R517A-R | GAAATCCGTGGCCTCTTCTGCCCTATCCAATGGCTT |
| Sir2E518A-F | AAGCCATTGGATAGGCGAGCAGAGGCCACGGATTTC |
| Sir2E518A-R | AATGAAATCCGTGGCCTCTGCTCGCCTATCCAATGG |
| Sir2R440A-F | CCCATTCTGCACCGTGTCGCGCATAGAACACATTAT |
| Sir2R440A-R | TGCATAATGTGTTCTATGCGCGACACGGTGCAGAAT |
| Sir2K360A-F | ATAACCTTCTCCGAGCTGGCGGAAAGGATAAGTCAG |
| Sir2K360A-R | TTTCTGACTTATCCTTTCCGCCAGCTCGGAGAAGGT |
| Sir2S357A-F | GCTAGTCCTATAACCTTCGCCGAGCTGAAGGAAAGG |
| Sir2S357A-R | TATCCTTTCCTTCAGCTCGGCGAAGGTTATAGGACT |
| Sir2S419A-F | GCCTCTACTTTCTTGAAAGCCTTTGTTGAGGAGGCA |
| Sir2S419A-R | AATTGCCTCCTCAACAAAGGCTTTCAAGAAAGTAGA |
| Sir2R105A- F | GATGATTACGAGGCGCAAGCAAAGTACCTTTTAGAA |
| Sir2R105A-R | GGCTTCTAAAAGGTACTTTGCTTGCGCCTCGTAATC |
| Sir2H163A- F | AAGCATTTGTCTGTTTATGCCCTTGAAGGATCCTAT |
| Sir2H163A-R | GGCATAGGATCCTTCAAGGGCATAAACAGACAAATG |
| Sir2K184A- F | GCCTTTCCCATTTACGCCGCGATTCATGGCGACTTC |
| Sir2K184A-R | ACGGAAGTCGCCATGAATCGCGGCGTAAATGGGAAA |
| Sir2R442A-R | TATAACTGCATAATGTGTTGCATGCCGGACACGGTG |
| Sir2R442A-F | CTGCACCGTGTCCGGCATGCAACACATTATGCAGTT |
| Sir2R529A-F | TTCATTCGAAGTCGAAGAGCTTATCGATTCAATCAA |
| Sir2R529A-R | AACATTGATTGAATCGATAAGCTCTTCGACTTCGAA |
| pAgoK170A-F | GACCTGCATGACCGAATAGCGGCGAAAGTTGCTCCG |
| pAgoK170A-R | GAGCGGAGCAACTTTCGCCGCTATTCGGTCATGCAG |
| pAgoF395A- F | AATCCGGGTCAACCGGTGGCCAAGGAAGCGGCTCTT |
| pAgoF395A- R | AGTAAGAGCCGCTTCCTTGGCCACCGGTTGACCCGG |
| pAgoY359A-F | CCTGTAGCTCCAGCATCAGCCCCAGTAGATAGAGGT |
| pAgoY359A- R | CAAACCTCTATCTACTGGGGCTGATGCTGGAGCTAC |
| Sir2D230A-F | CTATAGTGGTCGTGcTGAAAACGTTATGACTATGCTTCGGGCTGCAATCG |
| Sir2D230A-R | TAACGTTTTCAgCACGACCACTATAGCCTGATACAACAAG |
| Sir2H186AF | TTACGCCAAGATTGCTGGCGACTTCCGTTATCAAAAAATAA |
| Sir2H186AR | ACGGAAGTCGCCAGCAATCTTGGCGTAAATGGG |
| Sir2N142A-F | TTCACAACCGCCTTTGATGATGTCATAGAAACCGCCTTC |
| Sir2N142A-R | ATCATCAAAGGCGGTTGTGAAAACAACTTTGGTCTGGTTC |
| pAgo | TACTTCCAATCCAATGCCATGGCCGATAATCTTTCGCA |
| pAgo | CCTTCTTAAAGTTAAACAAAATTATTACATAAAAAACCGATAATCATATATTTCGTT |
| Sir2F | TGTTTAACTTTAAGAAGGAGATATACCATGGATGTCTTAACTGACAATGAG |
| Sir2R | TTATCCACTTCCAATGTTATTATCGGCCATATCCCACC |
